# Conjunctival HLA-DR and CD8 expression detected by impression cytology in ocular graft versus host disease

**Published:** 2013-07-19

**Authors:** Philipp Eberwein, Susanne Issleib, Daniel Böhringer, Hans Mittelviefhaus, Johannes Schwartzkopff, Juergen Finke, Thomas Reinhard

**Affiliations:** 1 University Eye Hospital, Albert-Ludwig-University, Freiburg, Germany; 2 Department of Hematology and Oncology, Albert-Ludwig-University, Freiburg, Germany

## Abstract

**Purpose:**

To assess the expression of human leucocyte antigen (HLA)-DR in epithelial cells and cluster of differentiation (CD8)-positive lymphocytes as possible markers of chronic ocular graft versus host disease (cGvHD) after hematological stem cell transplantation (HSCT).

**Methods:**

Twenty-seven consecutive patients with dry-eye symptoms following HSCT (24 [89%] with peripheral blood stem cell transplantation and 3 [11%] with bone marrow transplants; 17 [63%] familiar allogenic grafts) and 19 age-matched controls were included. Conjunctival impression cytology specimens were stained for HLA-DR, cytokeratin 19, and CD8. Oxford grading scale, blinking frequency, Schirmer test, tear film break-up time (TBUT), and Ocular Surface Disease Index (OSDI) were also recorded. Wilcoxon nonparametric testing was used to compare controls and HSCT recipients and to assess HSCT recipient subgroups with and without clinical cGVHD.

**Results:**

Eighteen patients showed clinical signs of ocular cGVHD. TBUT and Schirmer test scores were significantly lower in patients, while Oxford grades and OSDI were significantly higher than in controls. Epithelial HLA-DR expression was generally higher in HSCT recipients than in controls, but it did not correlate with ocular cGVHD status. CD8-positive lymphocytes were identified in five patients with ocular cGvHD and one control.

**Conclusions:**

A strong HLA-DR expression as detected by impression cytology appears to indicate a general HSCT response and fails to predict ocular cGVHD. However, the detection of CD8-positive lymphocytes using impression cytology was frequently associated with ocular cGvHD. Our data warrant further evaluation of CD8 expression in impression cytology, along with comparison to conjunctival biopsies and brush cytology, as impression cytology may offer a less invasive strategy for assessing cGVHD status.

## Introduction

Following hematopoietic stem cell transplantation (HSCT), 40% of patients develop systemic acute graft versus host disease, and 30%–70% develop systemic chronic graft versus host disease (cGvHD), which carries the risk of eye involvement [1,2]. Ocular cGvHD involves the lacrimal gland, the conjunctiva, and the meibomian glands and can progress to substantial destruction of these structures, causing severe dry eye. Depending on the severity of ocular involvement, patients complain about foreign-body sensation, reduced vision (due to corneal epithelial microdefects), and severe blepharospasm induced by extensive glare and light sensitivity [3].

However, not all patients show the same extent of dry eye after HSCT, and not all patients with dry eye following HSCT have an underlying ocular cGvHD [4]. For therapy and prognosis, the ophthalmologist needs to differentiate between “conventional dry eye” and “dry eye due to active ocular cGvHD.” Scarring of the tarsal plate, severe meibomian gland disease (MGD), loss of goblet cells, and infiltration of the basal conjunctival epithelium and the conjunctival stroma with CD8-positive cells are hallmarks of ocular cGvHD [5-7].

Recently, Rojas et al. examined human leucocyte antigen (HLA)-DR, which is part of the major histocompatibility complex II and is involved in antigen presentation to immune cells, in conjunctival biopsies and found increased expression after HSCT [5]. Several previous reports using impression cytology suggest that HLA-DR expression increases in conjunctival epithelial cells in dry-eye disease [8-14], after ocular surface burn [15], in inflammatory eye disease [16], and in Stevens Johnson Syndrome [17]. At present, data on the usefulness of impression cytology for the evaluation of conjunctival HLA-DR expression in patients following HSCT or on a possible correlation of HLA-DR expression with ocular cGvHD are lacking.

Currently, clinical diagnosis of ocular cGvHD relies on slit-lamp examination. The detection of cluster of differentiation (CD)4+ and CD8+ lymphocytes in conjunctival biopsies is the only objective diagnostic test available. Unfortunately, biopsy may severely impair the patient, induce conjunctival scarring, and cannot be repeated frequently to monitor disease progression. Impression cytology may serve as a safe alternative procedure to detect inflammatory cells and test for ocular cGvHD after HSCT. Therefore, it was our goal to study conjunctival epithelial HLA-DR expression and CD8+ cells in impression cytology specimens of HSCT recipients, to assess if impression cytology might replace conjunctival biopsy in the evaluation of ocular cGVHD.

## Methods

The study followed the tenets of the Declaration of Helsinki, and informed consent was obtained from all patients. After approval by the ethical commission of the University of Freiburg (vote no: 75/09_110257), we included 27 eyes of 27 patients with dry-eye symptoms after HSCT. Ocular cGvHD was diagnosed according to the consensus criteria for clinical trials in cGvHD [18]. All patients underwent ophthalmological examination later than 100 days after transplantation. Nineteen age-matched eyes of 19 healthy controls without dry-eye complaints served as the control group. Ocular Surface Disease Index (OSDI), tear film break-up time (TBUT), Schirmer test, a slit-lamp exam, and a medical history were performed in all subjects. The TBUT was recorded as the time interval between the last blink after fluorescein dye staining and the appearance of the first corneal black spot. The Schirmer test (baseline tear secretion) was performed with topical anesthesia instilled into the lower fornix 5 min before measurement. Filter paper strips (HS Clement Clarke International, Harlow, UK) were placed in the lower conjunctival fornix for 5 min, and the length of wet filter paper (in mm) was recorded. Blink rates were graded into normal versus elevated without blepharospasm and elevated with blepharospasm on the basis of the investigator counting seconds silently. The Oxford grading scale and the occurrence of scarring at the tarsal plate of the upper and lower eyelid were evaluated as well.

### Impression cytology

Impression cytology specimens were collected with topical anesthesia and before instillation of staining eye drops. Polyethersulphone filters (5–6; Suopor® 200 membrane, Pall Corporation, Ann Arbor, MI) were applied consecutively to the same area of unexposed superior bulbar conjunctiva. The filters were then transferred to tubes containing 300 µl of Dulbecco’s modified Eagle’s medium (DMEM; PAA, Cölbe, Germany) with 10% fetal calf serum (FCS; Serva, Heidelberg, Germany). Tubes were stored at 4 °C before cells were harvested from filter papers by agitating each tube for 30 min. Cell suspensions were spun onto a glass slide (300 ×g for 10 min) using a Shandon Cytospin 3 centrifuge (Thermo Fisher Scientific, Waltham, MA) and then dried and fixed with 5% ice-cold methanol/acetone for 5 min.

### Indirect immunofluorescence

All antibodies were adjusted to their final working dilution in phosphate buffered saline (PBS) containing 0.05% Tween-20 (Sigma, Munich, Germany) and 1% FCS. Slides were stained with primary antibodies against HLA-DR (mouse monoclonal antihuman; DAKO, Glostrup, Denmark; working dilution 1:50), cytokeratin (CK)19 (mouse monoclonal; Santa Cruz Biotechnology, CA; working dilution 1:100) and CD8 (mouse monoclonal; AbD Serotec, Duesseldorf, Germany; working dilution 1:50). After incubation with the primary antibody for 60 min, specimens were washed three times (5 min each) in PBS, followed by incubation with the secondary fluorochrome-conjugated antibody for 40 min (Alexa FluorTM 488; goat antimouse; working dilution 1:200 or Alexa FluorTM 594; goat antirabbit; working dilution 1:200; both from Invitrogen, Darmstadt, Germany). Nuclei were counterstained by DAPI (Sigma, Munich, Germany). After washing, specimens were embedded in mounting medium (Biomeda, Foster City, CA) and evaluated with a Keyence BZ-9000 fluorescence microscope (Keyence Germany, Neu-Isenburg, Germany). For negative and positive control of CD8, cytospins of peripheral blood leucocytes were stained. In case of negative controls, the secondary antibody was directly added without having used the primary antibody before.

### Quantification of HLA-DR- and CD8-expressing cells

**Figure 1:**
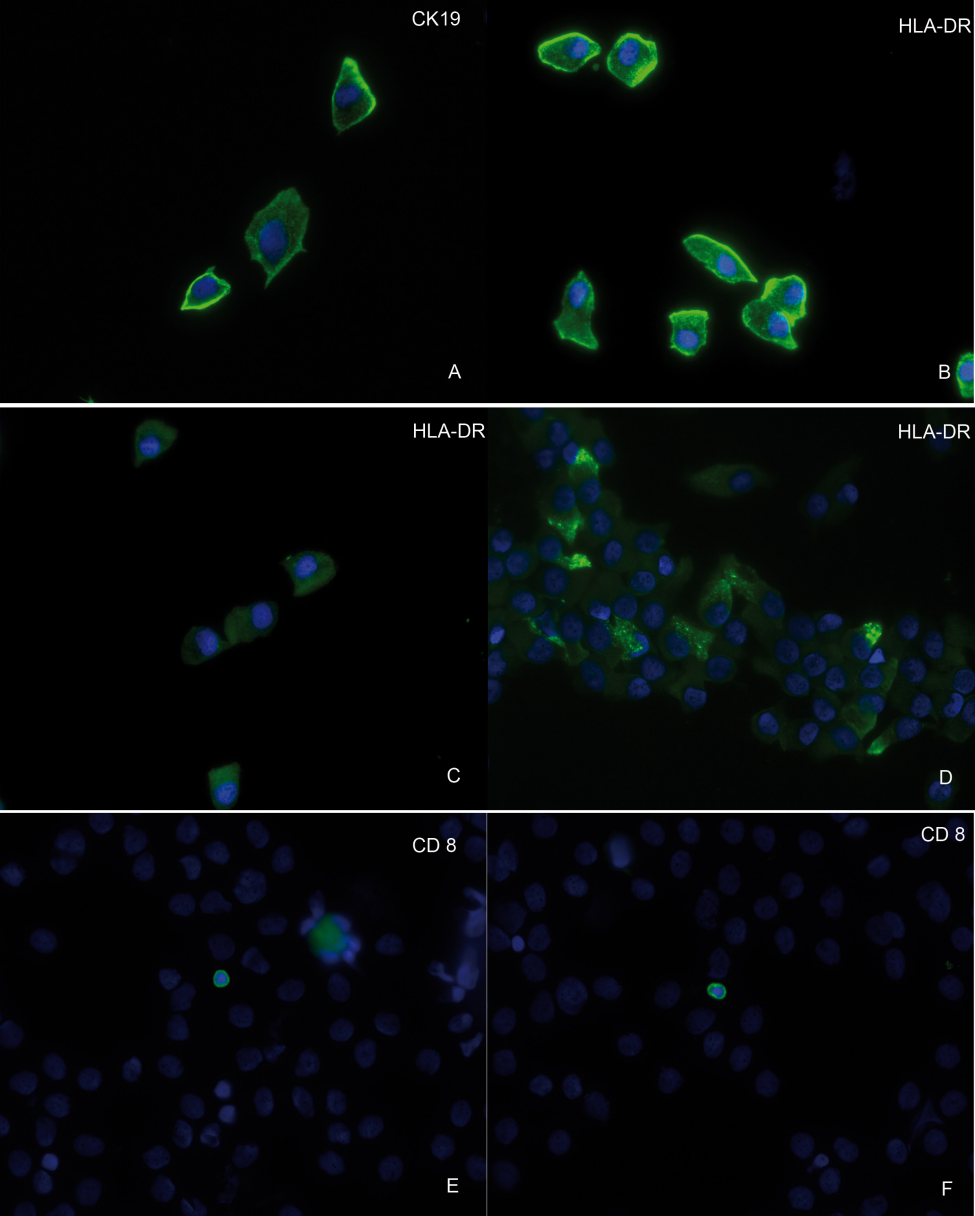
Immunofluorescence images of cytospins showing cytokeratin (CK)19-positive epithelial cells. **A**: Shown are examples of human leucocyte antigen (HLA)-DR-positive epithelial cells from different patients with ocular chronic graft versus host disease (cGvHD). **B**: Image shows single epithelial cells with strong HLA-DR expression. **C**: Depicted are single epithelial cells with low HLA-DR expression. **D**: Shown is an epithelial cell sheet with HLA-DR-negative and -positive epithelial cells. **E**, **F**: Displayed are two cluster of differentiation (CD)8-positive lymphocytes of patients with ocular cGvHD.

HLA-DR-positive epithelial cells were counted in ten different fields. Only cells with a typical epithelial morphology were analyzed. At least 50 cells were counted and the data expressed as percentages of positive cells. CD8-positive cells were identified by their CD8 expression and lymphocytic morphology (Figure 1E). The whole slide was screened for CD8-positive lymphocytes and counted as positive if at least one CD8-positive cell could be detected. Independent and blinded observers did all analyses.

### Statistical evaluation

Wilcoxon nonparametric testing was used to compare clinical and laboratory parameters between groups and for subgroup analysis of HLA-DR expression in patients with or without cGvHD. All statistical computations were performed with the R platform.

## Results

### General clinical data

The 27 HSCT recipients (ten females) had been treated for various underlying hematological diseases (Table 1). In total, 24 patients (89%) had received peripheral blood stem cell transplantation, while three patients had received bone marrow transplants. Seventeen (63%) patients had received grafts from family donors. Sixteen patients were still on systemic immunosuppressant therapy (mycophenolate, sirolimus, tacrolimus, cyclosporin), and 13 patients received low-dose systemic steroids (2.5–17.5 mg/d) when impression cytology was conducted. Patients had oral (11), gastrointestinal (5), liver (4), or cutaneous (22) cGvHD, diagnosed by the bone marrow transplant unit of the University of Freiburg.

**Table 1:** Clinical data of patients following hematopoietic stem cell transplantation (HSCT).

**Hematopoietic disease**	**Patients (n=27)**
AML	37% (10)
B-NHL	4% (1)
CLL	7% (2)
CML	15% (4)
MDS	19% (5)
Multiple Myeloma	4% (1)
T-cell lymphoma	15% (4)

**Transplant source**	
Bone marrow familiar-allogenic	7% (2)
Bone marrow foreign-allogenic	4% (1)
Peripheral stem cells familiar-allogenic	56% (15)
Peripheral stem cells foreign-allogenic	33% (9)
Sex-Missmatch	48% (13)

**Systemic GvHD**	
Liver	15% (4)
Intestine	19% (5)
Oral	41% (11)
Cutaneous	81% (22)
Ocular	67% (18)
Systemic Immunosuppression	56% (15)
Mycophenolate	7% (2)
Ciclosporin	26% (7)
Everolimus	15% (4)
Sirolimus	7% (2)
Systemic Steroids (2,5 - 17,5 mg prednisolone /d)	48% (13)

### Ocular clinical data

**Figure 2:**
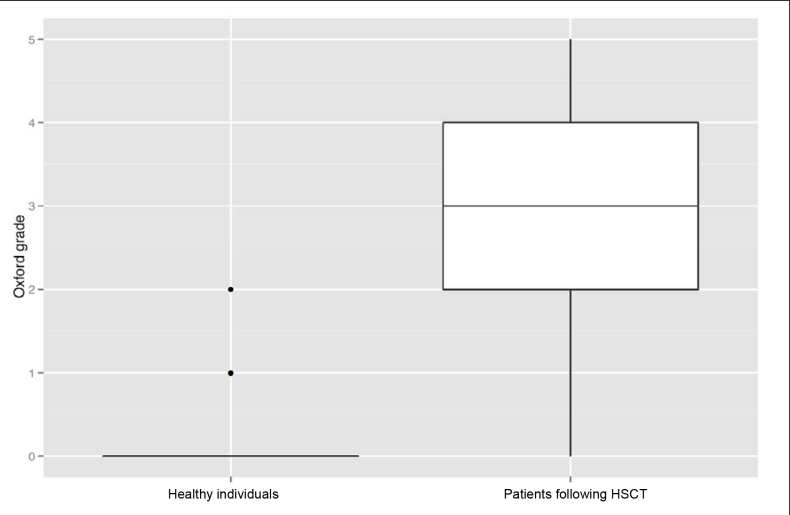
Oxford grades were compared between the control (n=19) and the patient group (n=27). Box plots display the median and quartiles (Q_0,25_ and Q_0,75_) and the total range. Median grade was 0 in the control group and 3 in the patient group, which was statistically significant (p<0.001, Wilcoxon non-parametric testing), showing that the patient group depicted significantly more ocular surface staining than the control group.

**Figure 3:**
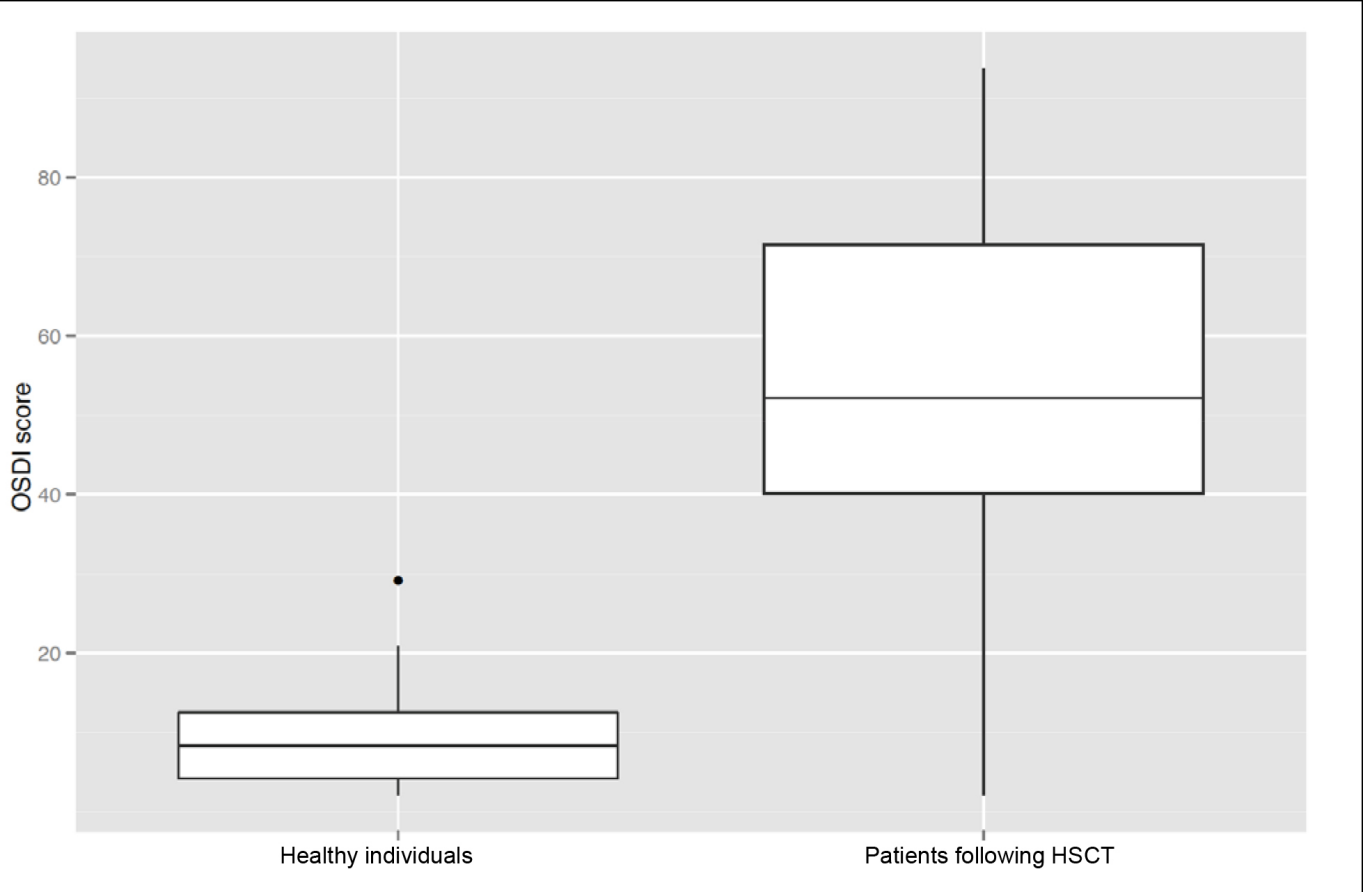
Box plots. Box plots display the median and quartiles (Q_0,25_ and Q_0,75_) and the total range of the ocular surface disease index score, which was 8.3 in the control group (n=19) and 52.2 in the patient group (n= 27; p<0.001, Wilcoxon non parametric testing), showing that the patient group had significantly more complaints from ocular surface disease than the control group.

Eighteen of 27 dry-eye patients following HSCT were diagnosed with ocular cGvHD according to the consensus criteria for clinical trials in cGvHD [18]. The mean Oxford grade was 3 in the patient group, indicating corneal epithelial alteration, while it was 0 in the control group (Figure 2). This finding was in accordance with an unstable tear film in the patient group as measured by the TBUT. A mean OSDI score of 52 in the patient group, compared to a mean score of 10 in the control group, confirmed this trend (Figure 3). Frequent and forced blinking was evident only in the patient group, accompanied by high sensitivity to light and glare. Patients also had significantly reduced Schirmer test results (Table 2). Table 3 provides a further comparison of clinical data between patients with and without ocular cGvHD. Only the Oxford grading scale and the blinking rate differed statistically significantly between both groups.

**Table 2:** Clinical data of controls in comparison to HSCT patients.

	Control (healthy individuals without dry eye complaints; n=19) (1^st^ quartile / median / 4^th^ quartile)	HSCT patients (n=27) (1^st^ quartile / median / 4^th^ quartile)	Level of significance (Wilcoxon test)
Schirmer test	0.25/5/10	0/0/2	p=0.005
Age	25/40/63,5	47/54/60	p=0.26
OSDI	4.2/8.3/12.5	40.1/52.2/71.6	p<0.001
Break-up time	3.5/8/9.5	3/3/4	p=0.005
Sex (female)	50%	37%	p=0.57
Oxford grading scale	0/0/1	2/3/4	p<0.001
Blinking rate=elevated with blepharospasm	11% (2)	93% (25)	p<0.001
% HLA-DR expression	3/5/11	20/29/42	p<0.001

Distribution of clinical values, human leucocyte antigen (HLA)-DR expression and cluster of differentiation (CD)8 expression between controls (n=19) and patients following hematopoietic stem cell transplantation (HSCT; n=27). Patients following HSCT expressed significantly more HLA-DR, had a lower Schirmer score and tear break-up time, a higher oxford grade and a higher ocular surface disease index score.

**Table 3:** Comparison of the clinical data of patients with and without ocular cGvHD following HSCT.

	HSCT patients without ocular cGvHD (n=9) (1^st^ quartile / median / 4^th^ quartile)	HSCT patients with ocular cGvHD (n=18) (1^st^ quartile / median / 4^th^ quartile)	Level of significance (Wilcoxon test)
Schirmer test	0/2/4	0/0/0.5	p=0.07
OSDI	31/51/56	43/54/77	p=0.15
Break-up time	2/3/4	3/3.5/4	p=0.52
Sex (female)	56% (5)	28% (5)	p=0.15
Age (years)	46/55/56	48/53/62	p=0.88
Oxford grading scale	1/2/3	2/3/4	p=0.04
Blinking rate=elevated with blepharospasm	78% (7)	100% (18)	p=0.03
% HLA-DR expression	13/29/44	22/28/41	p=0.88
CD8	11% (1)	22% (5)	0.484
Systemic immunosuppression	67%	33%	p=0.31
Conjunctival scar	33%	83%	<0.05
cGvHD skin	89%	78%	p=0.48

Comparison of the clinical data of patients with (n=18) and without (n=9) chronic ocular graft versus host disease (cGvHD) following hematopoietic stem cell transplantation: Statistical differences were found for Oxford grades, blinking rates and conjunctival scarring. 4 out of 5 patients with conjunctival cluster of differentiation (CD)8 positive lymphocytes suffered from ocular cGvhD.

### Immunofluorescence in impression cytology specimens

#### CK19 expression

To ensure that only conjunctival epithelial cells were analyzed, all specimens were checked for CK19 expression, a biomarker for conjunctival epithelial cells [19]. All cells that expressed CK19 showed an epithelial morphology as well (Figure 1A).

#### HLA-DR and CD8 expression

**Figure 4:**
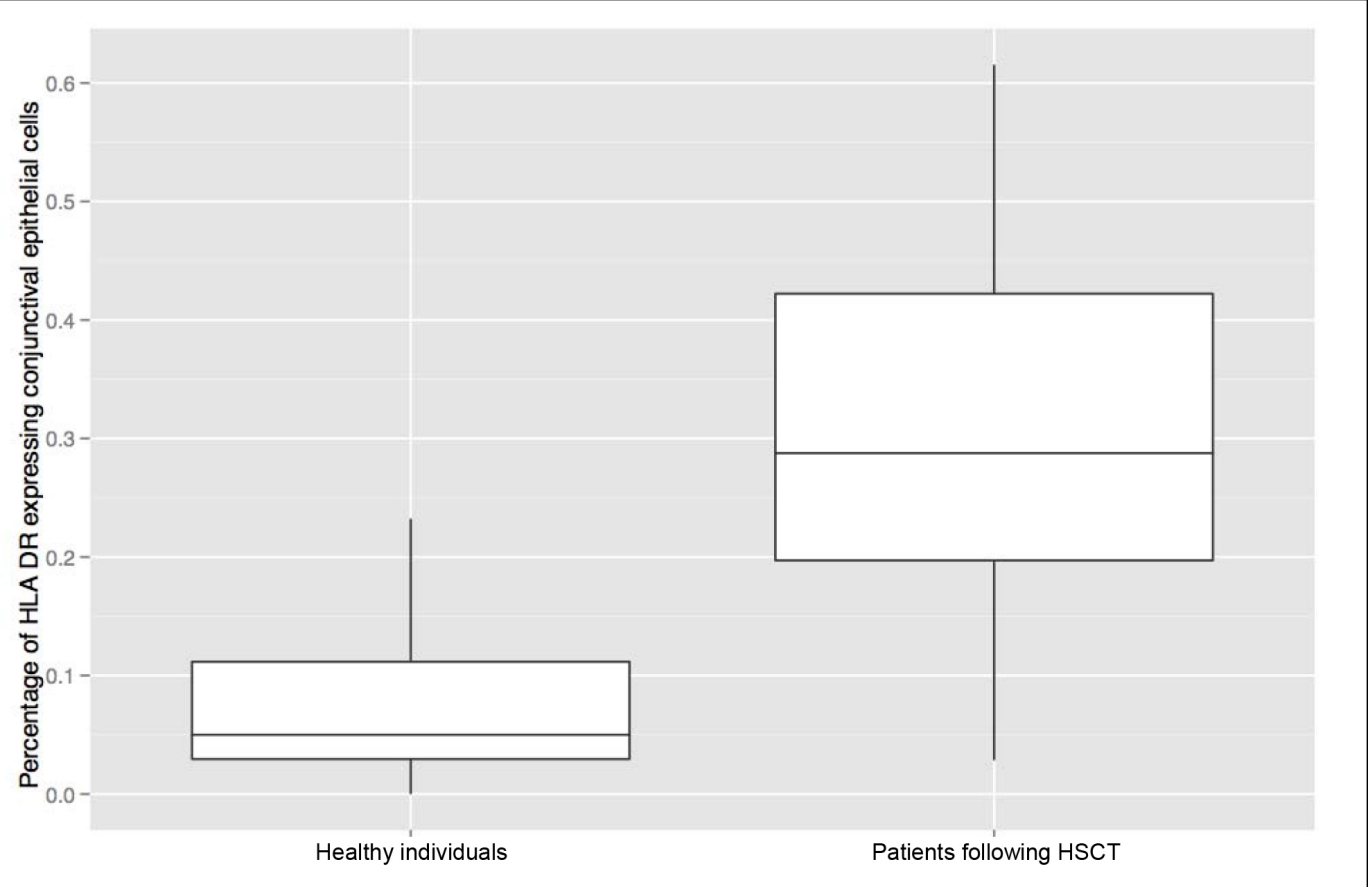
Box plots display the median and quartiles (Q_0,25_ and Q_0,75_) and the total range of human leucocyte antigen (HLA)-DR expression. A median of 5.0% of conjunctival epithelial cells expressed HLA-DR in the control group (n=19), while 29% of conjunctival epithelial cells expressed this marker in the patient group (n=27; p<0.001).

HLA-DR expression was localized to the cytoplasm and the plasma membrane of conjunctival epithelial cells (Figure 1B-D) and was significantly higher in patients following HSCT (Figure 4), suggesting an increased ocular surface inflammation in these patients this group (30.1% in the HSCT group versus 7.65% in the control group). HLA-DR expression did not significantly differ between patients with and without ocular cGvHD. We further assessed the correlation between HLA-DR and the ocular clinical data in a multiple linear regression model comprising patient age, Oxford grade, TBUT, and the OSDI score. Here, age (p<0.02) and TBUT (p<0.02) were statistically significantly correlated with the HLA-DR expression, whereas the Oxford scale and OSDI missed statistical significance.

CD8 expression was assessed in the specimen taken from the deepest conjunctival layer accessible by impression cytology. In most patients, this was the fifth impression cytology sample taken from the same area. We identified CD8-positive T cells in five patients (Figure 1E). Four out of these five patients also showed clinical signs of ocular cGvHD (Figure 1E). One control showed CD8-positive lymphocytes as well. Interestingly, patients with CD8-positive cells in impression cytology performed worse throughout the tests, with the exception of the OSDI. While statistical significance was not reached in all parameters, it appears that patients with CD8 cells in impression cytology exhibited significantly more ocular surface alteration (demonstrated in the Oxford test) than patients without CD8 cells. Median HLA-DR expression was higher in the group of patients with positive CD8 cells as well, without reaching significance (Table 4).

**Table 4:** Comparison of the clinical data of the 5 patients with positive CD8 cells in impression cytology and the other patients.

	Patients with positive CD8 cells (n=5)	Patients with negative CD8 cells (n=22)	Level of significance
Age	48/50/59	47/55/61	p>0.05
Oxford	4/4/5	2/3/3	p=0.01
Schirmer	0/0/0	0/0/2,25	p>0.05
OSDI	42/52/52	40/55/73	p>0.05
BUT	2/2/2	3/4/4	p=0.008
HLA-DR %	29/30/40	16/25/44	p>0.05
Blinking rate=elevated with blepharospasm	100%	91%	p>0.05
Sex mismatch	40%	50%	p>0.05
Clinical signs of ocular cGvHD	80%	64%	p>0.05

Comparison of the clinical data of the 5 patients with positive cluster of differentiation (CD)8 cells in impression cytology and the CD8-negative patients (n=27). These five patients performed worse in most of the tests with the exception of the ocular surface disease index. Statistical significance was reached in the Oxford test. Median human leucocyte antigen (HLA)-DR expression was higher in CD8-positive patients as well, without reaching significance.

## Discussion

Although our sample size was fairly small, it is comparable to previous studies [5] and is limited by the rarity of clinically active ocular GvHD. In our series of 27 dry-eye patients following HSCT, 63% showed signs of ocular surface involvement in cGvHD, similar to previous observations [4-6,20-22]. Besides strong ocular surface staining, conjunctival scarring of the tarsal plate and inflammatory changes of the lid margin, increased blinking frequencies and photophobia were the most characteristic symptoms of severe ocular cGvHD. Patients with accentuated blinking and photophobia showed the highest OSDI scores, indicating severely impaired quality of life. Overall, the OSDI correlated well with the severity of ocular surface involvement, which is in agreement with recent publications [21,23,24].

Interestingly, although the Schirmer test showed significant differences between controls and the patient group, some controls exhibited low Schirmer results without ocular surface changes under slit-lamp examination or signs of impairment in the OSDI. This is in line with results from two other studies, which suggested a possible overestimation by the Schirmer test in the diagnosis of ocular cGvHD [18,21,25]. Other studies questioned the reproducibility and sensitivity of the Schirmer test in Sjögren’s syndrome and proposed the use of the anterior segment OCT as an alternative for quantification of the tear meniscus [26]. It appears that methods with greater robustness may be needed to quantify the aqueous phase of the tear film in ocular cGvHD. In light of the strong inflammatory component of ocular cGVHD, markers for ocular inflammation should be included in the diagnostic evaluation. This study represents a first step in this direction by evaluating HLA-DR expression and CD8+ cells in impression cytology specimens of HSCT recipients with severe dry-eye symptoms.

Definite diagnosis and monitoring of ocular cGVHD is challenging, due to a lack of objective criteria. Besides slit-lamp examination and clinical tests for dry eye, conjunctival biopsy is the only option for substantiating the diagnosis of ocular cGvHD [6]. Unfortunately, conjunctival biopsy is potentially harmful for these patients and cannot be repeated indefinitely if disease monitoring is required. Impression cytology may serve as an alternative. It has been increasingly performed in dry eye to monitor ocular surface inflammation using immunofluorescent staining for HLA-DR [9,11,13,27], and this technique offers the possibility of repeated examinations without harming the patient and inducing a conjunctival inflammation. In contrast to other studies, we decided to use cytospins and a microscopic analysis of the stainings. The main reason for this decision was the difficulty of loosening cells from the membrane, resulting in significant cell loss for analysis. Cell loss also increases from using several centrifugation steps regarding the serum contents in the fluid [9]. Further uncertainty in flow cytometry may result from the compensation process, which may be investigator-dependent when done by hand or incorrectly performed when done by the machine itself. Therefore, we decided to use cytospins with impression cytology in our study.

Using impression cytology, we detected a significantly elevated HLA-DR expression in HSCT patients compared to healthy individuals. Using a far less invasive approach than a conjunctival biopsy, these data confirm the results of a previously published study by Rojas et al. that found increased HLA-DR expression in histological sections of patients following hematological stem cell transplantation (HSCT) [5]. Rojas et al. described increased HLA-DR expression following both autologous and allogenic HSCT. The authors of that manuscript differentiated patients with and without ocular GvHD according to the Schirmer results, as defined by the consensus conference [18]. While the authors of that study found that HLA-DR expression was higher following autologous than following allogenic HSCT, an analysis of epithelial HLA-DR expression with respect to the presence or amount of active ocular GvHD in patients was not provided. In contrast to that study, one major goal of our study was to look for a correlation of the amount of ocular GvHD and the amount of epithelial HLA-DR expression, to help clinicians with diagnosis. This is a factor that was not addressed by the previous studies. Moreover, we used a different approach with impression cytology to overcome the problems and discomfort of performing a conjunctival biopsy in these patients. Furthermore, increased HLA-DR levels in conjunctival epithelial cells have been reported in various other ocular surface diseases, such as dry eye, Sjögren’s syndrome, chronic unpreserved glaucomatous eye-drop use, and allergic conjunctivitis [13,28,29]. Interestingly, only one previous study suggested a correlation of HLA-DR expression, slit-lamp examination, and ocular surface scores [14]. While our data indicate an increased HLA-DR expression following HSCT and a correlation of HLA-DR with TBUT and age in a multiple linear regression model, it fails to indicate a clear correlation of HLA-DR expression and the severity of ocular cGvHD. This may be for several reasons: First, although we tested all patients beyond 100 days of HSCT, the time point of impression cytology varied between patients, and timing may be critical for the amount of detectable HLA-DR expression. Second, some patients were under systemic immunosuppressants, which may alter HLA-DR expression. Third, it is known that other factors in dry eye may upregulate HLA-DR expression as well: Many patients following HSCT show an increased tear osmolarity [30,31], and Versura et al. demonstrated that an increase in tear osmolarity upregulates conjunctival HLA-DR expression in vivo and in vitro [32].

Wang et al. previously described a decrease in goblet cells and a general increase in inflammatory cells with increasing severity of dry eye in patients following HSCT [4]. Others, as well as our own previous studies revealed that CD25, CD68, CD1a, and CD8 were among the predominant inflammatory cells in ocular cGvHD, with CD8-positive lymphocytes being the most specific for ocular cGvHD [5,6]. In the present study, we found CD8-positive lymphocytes in five HSCT patients and in one control, using impression cytology. Four patients with CD8-positive cells were classified as having ocular cGvHD on the basis of their clinical presentation. Although we might have missed CD8-positive cells due to their occurrence in deeper conjunctival layers [6,33], the detection of these lymphocytes by impression cytology may strengthen a clinical suspicion of ocular cGvHD. In addition, a comparison of patients with and without CD8-positive cells showed that CD8-positive cells could be an indicator of more severe dry eye. On the other hand, we found CD8-positive cells in one control, as well, which is in accordance with recently published data showing that CD8-positive T cells occur in the conjunctiva of healthy individuals, as well as in the conjunctiva of Sjögren’s patients [34,35]. These studies used brush cytology, which is more invasive than impression cytology. Therefore, the detection of immune cells, located in the basal epithelium or in the conjunctival stroma, is more likely and could be a reason for the lower detection rate in our study. However, studies including a larger number of patients, as well as brush and impression cytology, are needed to test for more subsets of lymphocytes, predict a CD4/CD8 ratio, and define the sensitivity and specificity of the test in comparison to brush cytology and conjunctival biopsy.

In summary, our data indicate that conjunctival HLA-DR expression is significantly increased in patients following HSCT compared to healthy individuals. In our cohort, this expression did not correlate with the presence of ocular cGvHD but could represent a general ocular surface inflammation occurring following HSCT, or could be attributed to the dry-eye situation in these patients. Impression cytology also allowed detecting CD8-positive lymphocytes in patients following HSCT with ocular cGvHD. This approach may be a future option for clarifying a clinical suspicion of ocular cGvHD and may be supplemented by other markers and techniques to specify the intraepithelial lymphocyte population in patients with ocular cGvHD. Of all clinical scores evaluated in this study, the OSDI proved to be the most valuable tool for follow-up in patients following HSCT, due to its good correlation with the degree of ocular surface involvement.
